# Empowering patients through AI: the role of ChatGPT in daily monitoring of blood pressure and blood glucose levels

**DOI:** 10.21542/gcsp.2024.56

**Published:** 2024-12-31

**Authors:** Shrishti P. Khetan, Shruti Suresh Suvarna

**Affiliations:** American University Of Barbados Bridgetown, Saint Michael Barbados

Dear Editor,

Conversational AI, specifically ChatGPT, in has enormous potential in the daily monitoring and management of chronic disorders such as hypertension and diabetes mellitus. These conditions have become prevalent and significant health risks, requiring innovative tools to aid in self-care.

AI has become a valuable tool in healthcare, with ChatGPT offering patient support and tracking solutions. Designed by OpenAI, ChatGPT is an advanced language model that comprehends and generates human-like text and is competent in engaging in natural discussions, responding to queries, and providing detailed explanations on various topics^1^. By leveraging natural language processing and machine learning, ChatGPT can assist patients in managing their health more effectively as a virtual assistant^2^.

Hypertension impacts over 1.3 billion individuals globally, and diabetes mellitus affects 537 million adults worldwide, requiring constant monitoring to lower health risks. However, patients struggle with daily tracking and self-management due to complexity and time management^3^.

Self-management is crucial in managing chronic illnesses, involving specific measures, regular follow-ups, and emotional health management. Due to the complexity and potential incompatibility with other healthcare recommendations, patients find it difficult to commit to these tasks daily.

AI-powered virtual medical assistants (VMAs) have seen a surge in integration within healthcare systems. Organizations like Onduo utilize technology for virtual coaching, offering tailored advice through mobile applications based on inputs like food intake, glucose levels, and activity tracking^4^. Omada Health and HealthifyMe exemplify AI-driven approaches with proven benefits in chronic disease management. Omada Health’s behavior change programs enhance adherence and reduce chronic disease risk factors through personalized interventions^5^. HealthifyMe leverages real-time data analysis to provide actionable insights, helping users manage their health proactively^6^. Leitner et al. (2022) also demonstrated that an AI-driven lifestyle intervention platform led to significant blood pressure reductions, with experimental group participants experiencing average reductions of 4.0 mmHg systolic and 4.7 mmHg diastolic^7^.

ChatGPT can be linked to scheduling apps like Google Calendar to provide personalized reminders for monitoring blood pressure, glucose levels, and medication, improving patient compliance. It can analyze data to account for variations caused by diet, stress, or medication and offer quick, precise summaries and patient statistics. [Table 1 shows a monthly summary of patient trends and recommendations, and Figure 1 shows an example of a reminder ChatGPT can sent to patients].

**Table 1:** Monthly summary of Patient trends and recommendations.

Monthly Health Summary: July 2024							
Patient: John Doe:							
Health Log For July:							
DATE	Avg Bp (mmHg)	Fasting Glucose (mg/dl)	Postprandial Glucose (mg/dl)	Medication adherence	Diet Notes	Stress Level (1-10)	Notes
July 1	132/85	118	165	Yes	Balanced, dessert after dinner	5	Slightly stressed
July 2	128/82	120	158	Yes	Skipped lunch, heavy dinner	6	Busy day at work
July 3	130/80	115	160	Yes	Light meals, low sugar intake	4	Managed stress well
July 4	135/88	125	170	Yes	Party, high sugar intake	7	Social stress, work deadlines
July 5	129/83	119	155	Yes	Normal diet	5	Moderate
July 6	133/87	122	165	Yes	High-carb meal for dinner	6	Busy day
July 7	131/84	121	162	No	Missed Breakfast,moderate sugar	6	Family stress
July 8	127/81	116	157	Yes	Well-balanced diet	3	Low
July 9	130/82	119	160	Yes	Skipped lunch,Healthy dinner	5	Moderate
July 10	132/85	118	164	Yes	Balanced diet	5	Moderate
……	……..	………	………	…….	…….	……	…….

**Figure 1.**
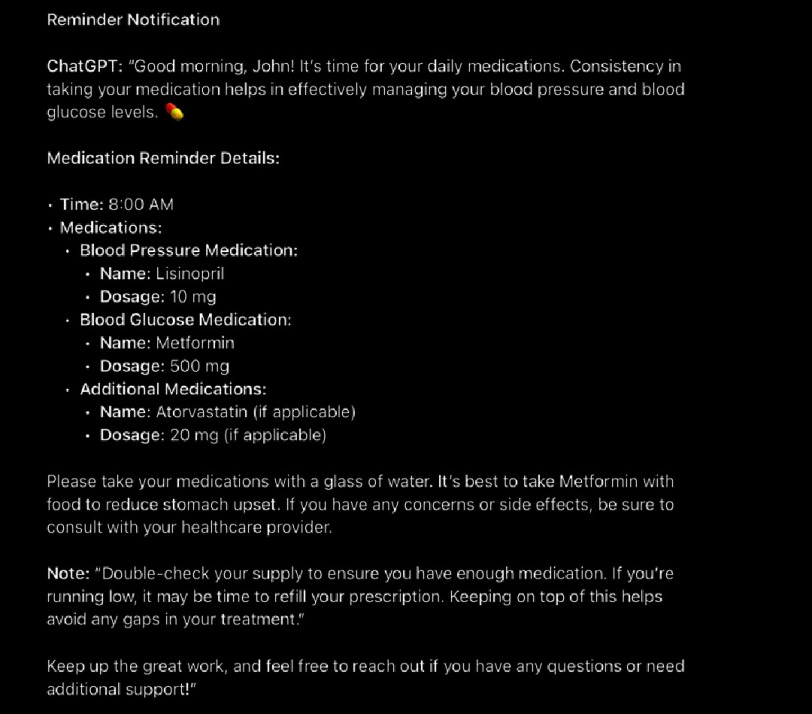
A screenshot of a sample reminder for the patient.

Generated Response Based on Data:

## ChatGPT:

“John, reviewing your data for July, here are some key observations and recommendations:

*Blood Pressure Trends:*

Your blood pressure readings averaged around 130/84 mmHg, with a few higher readings on days marked with higher stress levels, such as July 4th. It’s important to manage stress to help keep your blood pressure in check.

*Blood Glucose Levels:*

Your fasting glucose levels have been relatively stable, averaging 120 mg/dL, but there were occasional spikes in postprandial readings, particularly after high-sugar intake on social occasions. Aim to moderate your sugar intake during such events.

*Medication Adherence:*

Excellent adherence, with only one missed dose on July 7th. Maintaining this consistency is crucial for managing your conditions.

*Diet and Lifestyle:*

Your notes indicate some days with skipped meals or high-carb intake, which can influence both glucose levels and overall energy. Consistency in meals, focusing on balanced, low-sugar options, will be beneficial.

*Stress Levels:*

Stress levels fluctuated, with higher levels during busy workdays and social events. Incorporating relaxation techniques, like short breaks or mindfulness exercises, can help manage stress more effectively.

## Recommendations:

Diet: Continue with a balanced diet, and be mindful of sugar intake, especially during social events. Try not to skip meals as this can affect blood glucose stability.

Stress Management: Explore stress-reducing activities that fit your schedule, such as a short walk, deep breathing exercises, or meditation.

Routine Monitoring: Keep up the good work with medication adherence and daily monitoring. Consistent tracking helps in understanding patterns and making informed decisions.

### Follow-Up

• It would be helpful to schedule a follow-up with your healthcare provider to discuss these observations and make any necessary adjustments to your treatment plan.

Table of Key Metrics:

**Table:** 

Metric	Value
Average Blood Pressure	130/84 mmHg
Average Fasting Glucose	120 mg/dL
Average Postprandial Glucose	160 mg/dL
Medication Adherence	95%
Average Stress	5

This summary provides a comprehensive overview of your health trends and offers actionable insights to support your self-management efforts. Keep up the great work, and don’t hesitate to reach out if you have any questions or need further guidance.”

Moreover, ChatGPT can provide specific recommendations for medication compliance and lifestyle changes, including diet and exercise, promoting the self-management of chronic conditions. It answers most patient queries with reasonable, analyzed information and clarifies patient questions concerning their health condition. ChatGPT can also send real-time alerts based on patient data, such as spikes in readings, enabling patients to act promptly to avoid complications and improve their health. This feature enhances care quality by freeing time and improving the quality of care. Studies have highlighted ChatGPT’s capabilities in natural language processing can enhance chronic condition management by supporting patient education and communication. However, its dependence on textual data and absence of direct computational analysis can limit its ability to accurately interpret complex health data necessitating further validation for interpreting health data variations. Ethical, privacy, and contextual challenges must also be considered when integrating ChatGPT into chronic condition care^8^.

While ChatGPT significantly improves patient participation and compliance, its efficacy is limited by the quality of its training data, which can cause inaccuracies and biases in recommendations. Unclear queries can generate repetitious and unrelated replies, leading to misunderstandings. Data privacy is also a significant concern, especially since handling health data requires stringent protection standards and policies Compliance with healthcare data protection regulations, such as HIPAA and GDPR, is essential. Robust security measures must be implemented to safeguard sensitive health information, including end-to-end encryption and secure data storage. Additionally, data ownership should be clearly defined, ensuring patients retain control over their health data and are informed about its usage^9^.

In conclusion, ChatGPT dramatically improves patient participation and compliance with recommended health management regimens and health results. Despite its usefulness, it should not be considered professional medical guidance. Investigations on AI’s ethical analysis and incorporation in healthcare systems remain paramount, focusing on model accuracy, minimizing biases, and ensuring data privacy. Ethical frameworks are essential for responsibly integrating AI into healthcare systems, maximizing its positive impact while addressing potential risks.

We hope this letter supports the perspective of AI in managing chronic diseases and we look forward to discussing the future implementation of these technologies to enhance patient.
